# Dialectical nursing care: a concept analysis

**DOI:** 10.17533/udea.iee.v43n2e04

**Published:** 2025-07-23

**Authors:** Julia Valeria de Oliveira Vargas Bitencourt, Marcela Martins Furlan de Leo, Adriana Remiao Luzardo, Priscila Biffi, Dilzilene Cunha Sivirino Farias, Jeferson Santos Araújo

**Affiliations:** 1 Nurse, Ph.D. Email: julia.bitencourt@uffs.edu.br. https://orcid.org/0000-0002-3806-2288 Universidade Federal da Fronteira Sul Brazil julia.bitencourt@uffs.edu.br; 2 Nurse, Ph.D. Email: marcela.leo@uffs.edu.br. https://orcid.org/0000-0003-3457-5999 Universidade Federal da Fronteira Sul Brazil marcela.leo@uffs.edu.br; 3 Nurse, Ph.D. Email: adriana.luzardo@uffs.edu.br. https://orcid.org/0000-0002-9240-0065 Universidade Federal da Fronteira Sul Brazil adriana.luzardo@uffs.edu.br; 4 Nurse, M.Sc. student. Email: priscilabiffi99@gmail.com. Corresponding author. https://orcid.org/0000-0001-5476-5840 Universidade Federal da Fronteira Sul Brazil priscilabiffi99@gmail.com; 5 Nurse, M.Sc. Email: lenenfermeira@gmail.com. https://orcid.org/0000-0002-7408-1988 Universidade Federal do Espírito Santo Brazil lenenfermeira@gmail.com; 6 Nurse, Ph.D. Email: jeferson.araujo@uffs.edu.br. https://orcid.org/0000-0003-3311-8446 Universidade Federal da Fronteira Sul Brazil jeferson.araujo@uffs.edu.br; 7 Federal University of the Southern Frontier (UFFS), Chapecó/SC, Brazil Universidade Federal da Fronteira Sul Federal University of the Southern Frontier (UFFS) Chapecó/SC Brazil; 8 Federal University of Espírito Santo (UFES), Vitória/ES, Brazil Universidade Federal do Espírito Santo Federal University of Espírito Santo (UFES) Vitória/ES Brazil

**Keywords:** nursing care, nursing, concept formation, social determinants of health, practice patterns, nurses, nurses., atención de enfermería, enfermería, formación de concepto, determinantes sociales de la salud, pautas de la práctica en enfermería, enfermeros., cuidados de enfermagem, enfermagem, formação de conceito, determinantes sociais da saúde, padrões de prática em enfermagem, enfermeiros.

## Abstract

**Objective.:**

To analyze how the concept of dialectical nursing care is introduced in the scientific production of nursing.

**Methods.:**

It is a concept analysis based on Rodgers’ evolutionary model. An integrative literature review was carried out for the identification and selection of articles, in January 2022 and updated in March 2024, limited to the period between January 2010 and December 2023. The search was conducted in six databases: PubMed, Web of Science, Embase, Science Direct, Scopus and LILACS, combining the descriptors Dialectics, Health and Care.

**Results.:**

Based on Rodgers’ evolutionary model, it was possible to identify the attributes of the concept, which are: dialectical sensitivity, dialectical attitude, ambience and social determinants. The antecedents: The being and its social relationships, Health services, Work processes and Formative paradigm. The consequents: The being who cares for and the being cared for in their relationships, Work process and Formative paradigm.

**Conclusion.:**

This study contributed to the clarification of the concept that proposes the analysis of dialectics in the social production of illness and health, operating syntheses and new syntheses, in order to overcome the contradictions that historically cross people, macrostructure and area of nursing knowledge.

## Resumen

**Objetivo.:**

Analizar cómo se presenta el concepto de cuidados dialécticos de enfermería en la producción científica enfermera.

**Método.:**

Se trata de un análisis conceptual basado en el modelo evolutivo de Rodgers. Se realizó una revisión bibliográfica integradora para identificar y seleccionar los artículos en enero de 2022 y actualizada en marzo de 2024, limitada al periodo comprendido entre enero de 2010 y diciembre de 2023. La búsqueda se realizó en seis bases de datos: PubMed, Web of Science, Embase, Science Direct, Scopus y LILACS, combinando los descriptores Dialéctica, Salud y Cuidados.

**Resultados.:**

A partir del modelo evolutivo de Rodgers, fue posible identificar los atributos del concepto: sensibilidad dialéctica, actitud dialéctica, ambiente y determinantes sociales. Los antecedentes: el ser y sus relaciones sociales, los servicios de salud, los procesos de trabajo y el paradigma de formación. Los consecuentes: el ser que cuida y el ser cuidado en sus relaciones, el proceso de trabajo y el paradigma de formación.

**Conclusión:**

. Este estudio ha sido un aporte significativo al esclarecimiento del concepto que propone el análisis de la dialéctica en la producción social de la enfermedad y de la salud. El estudio ha mostrado que es posible producir nuevas síntesis que logran superan las contradicciones que atraviesan históricamente a las personas, la macroestructura y al área de conocimiento de enfermería.

## Resumo

**Objetivo.:**

Analisar como o conceito de cuidado dialético de enfermagem é apresentado na produção científica de enfermagem.

**Métodos.:**

Trata-se de uma análise de conceito baseada no modelo evolucionário de Rodgers. Realizou-se uma revisão integrativa da literatura para a identificação e seleção dos artigos, em janeiro de 2022 e atualizado em março de 2024, limitada ao período entre janeiro de 2010 e dezembro de 2023. A busca foi realizada em seis bases de dados: PubMed, Web of Science, Embase, Science Direct, Scopus e LILACS, combinando-se descritores Dialética, Saúde e Cuidado.

**Resultados.:**

Com base no modelo evolucionário de Rodgers foi possível identificar os atributos do conceito são: sensibilidade dialética, atitude dialética, ambiência e determinantes sociais. Os antecedentes: o ser e suas relações sociais, serviços de saúde, processos de trabalho e paradigma formativo. Os consequentes: o ser que cuida e o ser cuidado em suas relações, processo de trabalho e paradigma formativo.

**Conclusão.:**

Este estudo contribuiu para o esclarecimento do conceito que propõe a análise da dialética na produção social do adoecimento e da saúde, operando sínteses e novas sínteses para superação das contradições que atravessam historicamente pessoas, macroestrutura e área de conhecimento da enfermagem.

## Introduction

The concept of care is broad, formulated and discussed in various contexts. This understood as an act of caring for oneself and the other shows the influence of hermeneutics as an opportunity for care dialogs among the subjects, as an ethical and shared expression of knowledge and experiences of the health process.^1^ Concept analyses have helped nurses to understand care, deepening the reflection on looking at the other, such as the concept analysis of humanistic care in nursing. Research that adopted Rodgers’ Evolutionary Model^2^ highlighted some characteristics that translate a humanistic care environment, such as an environment capable of promoting healing, preserving human dignity, encouraging the development of talents and personal growth of the subjects. The concept of humanistic care in nursing proved to be useful in the construction of knowledge, instrumentation, planning and elaboration of guidelines and interventions for clinical management. 

Contemporaneously, we are faced with a broadening of the conception of health and care of a social nature. Historically, public health is a field under reconstruction in which philosophical aspects need to be critically revisited in terms of technological and specialized advances in health care, which is insufficient in terms of the need to reduce vulnerabilities and promote health and surveillance, requiring renewal in health practices.^3^^,^^4^ Thus, when considering the urgent need for the renewal of health practices, the concept of individual and collective, family and community care is influenced by historical dialectical conceptions, which are based on the paradigm of social production in health. Dialectical historical materialism, as a philosophical and theoretical framework, examines the work processes that display the conditions in which work develops in social life, as well as social relationships in the context of capitalism.^5^

Therefore, the mode of thought inscribed in dialectical historical materialism requires health professionals to pay attention to the events of the material reality of social life in a time frame, as each historical moment will reproduce a specific social history. It requires the professional to understand that social life is full of contradictions and that these contradictions are dynamic.^5^ Thus, this understanding of the world and reality is particularly applicable to nursing, with regard to its own origin and historical structuring of its workforce and the social production of care, which are aligned with these concepts, since it is in the possibility of contextualizing the dialectical historicity intrinsic to the health and disease process that the potential paradigmatic change in the health model is glimpsed. This theoretical framework, which influences some health systems in their epistemological origin, justifies the formulation of dialectical nursing care. In this sense, the current study aimed to analyze how the concept of dialectical nursing care is introduced in the scientific production of nursing, using Rodgers’ evolutionary method of conceptual analysis. 

## Methods

Study design. Concept analysis based on Rodgers’ evolutionary method, which consists of an inductive and descriptive study to outline the knowledge constructed on a concept. The six methodological steps formulated by Rodgers^6^ were used: 1) Definition of the concept of interest; 2) Identification and selection of the field for data collection; 3) Formulation of the attributes of the concept and contextual bases (antecedents and consequents); 4) Analysis of the characteristics of the concept (related concepts, for example); 5) Identification of an example concept (if appropriate); 6) identification of the implications for the development of new concepts.

Data collection period. The search was conducted in January 2022 and updated in March 2024, limited to the period between January 2010 and December 2023.

Selection criteria. Original articles, in Portuguese, English or Spanish, with different methodological approaches, were included. Conversely, articles that did not show the constituent elements of dialectical nursing care, publications outside the area of nursing knowledge, literature considered gray, editorials, letters to the editor, reflective and review studies were excluded.

**Figure 1:**
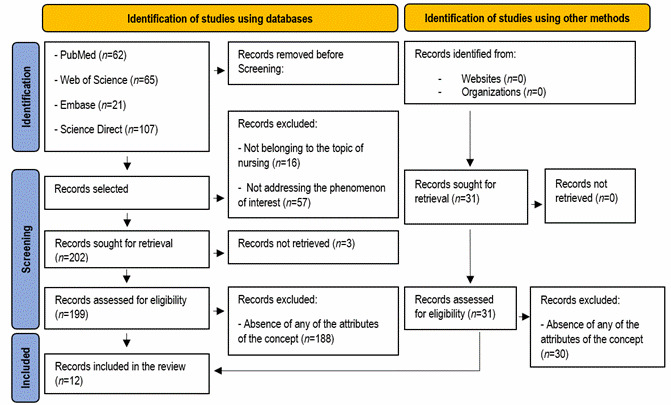
Representative data collection flowchart for concept analysis

Definition of the sample and instruments used. A total of 294 articles were identified: 62 in PubMed, 65 in Web of Science, 21 in Embase, 107 in Science Direct, 38 in Scopus and one in LILACS, and 31 references were identified in an unsystematic way, based on studies referenced in other articles. Accordingly, a total of 325 publications were selected. Two researchers, independently (first and last researcher), conducted the selection of studies through the Rayyan® platform.^7^ The process was blinded, and then possible disagreements resolved by consulting a third reviewer. The identified records were uploaded to the platform and, after the removal of the duplicate studies, 275 were analyzed, based on the reading of the titles and abstracts. The selection criteria were applied, with 73 records excluded: 16 were not from nursing and 57 did not address the phenomenon of interest. A total of 199 records were chosen for the systematic search and 31 for the unsystematic search, which were analyzed in full; and, among these, 218 were excluded because they did not show some of the attributes of the concept and its contextual bases (antecedents and consequents). For the final synthesis, 12 studies were selected.^8^^-^^19^ The PRISMA^20^ flowchart describes the selection of studies included in the concept analysis, as displayed in Figure 1.

Data collection. In order to identify the eligible studies in the area of nursing knowledge, an integrative review was carried out by two independent researchers.^21^ The search strategy was developed by combining the terms of MeSH, EMTREE and DeCS, such as Dialectics, Health and Care, as well as their synonyms and keywords, such as Dialog, Discussion, Well-being, Health Care, Prevention and Health Care, combined with the Boolean operators OR and AND. 

Data processing and analysis. The MaxQDA® version 2020^22^ software was used to determine the relationship between the contents that make up the concept, analyzing its characteristics from a broad perspective. The software allowed for a deeper study, exploring the extracted contents at two different levels of connected analyses: (1) the thematic area and its possible related themes: its representation in quantitative terms (frequency of coded segments) and qualitative (meaning transmitted in the context of dialectical nursing care) and (2) the similarities and differences contained in the perspectives addressed in the articles. These characteristics allowed us to synthesize the attributes that make up the concept and its contextual bases (antecedents and consequents). Subsequently, the elements were analyzed through inductive thematic analysis.^23^ Thematic analysis reports patterns among the data, allowing the grouping and interpretation of the various aspects of the concept. Thus, the themes representative of the antecedents, attributes and consequents of the concept were elaborated. It is noteworthy that this analysis process was not applied to the substitute and related terms.

## Results

Among the sets of analyzed evidence, six were published in Brazil,^8^^,^^10^^,^^11^^,^^14^^,^^15^^,^^18^ two in the United Kingdom,^9^^,^^17^ one in the USA,^13^ one in Canada,^12^ one in Norway^19^ and one in Vietnam^16^, with one in 2010,^18^ two in 2011,^14^^,^^19^ one in 2012,^17^ three in 2015,^9^^,^^15^^,^^16^ one in 2016,^8^ one in 2017,^12^ two in 2018^10^^,^^11^ and one in 2019.^13^ The concept of dialectical nursing care is complex and surrounded by other interconnected terms. 

Based on the analysis process, 11 themes were constructed, four related to antecedents, four to attributes and three to consequents, as follows: 

Antecedents. (i) *The being cared for and its social relationships*: Socio-historical conditions and contradictions that marginalize/segregate/stigmatize translate into social determinants of health that make individuals and groups vulnerable and affect their health status and social integration and the use of the health service structure;^15^^,^^21^^,^^42^ (ii) *Health services* (Care supply structure): State of programmatic vulnerability that generates the need to change the service structure to offer care actions due to stressors, panopticons and contradictions, as well as the need to maintain safety in the face of the variability of care shown in health services;^5^^,^^19^^,^^20^^,^^34^^,^^42^ (iii) *Work process* (work relationships/organization): Hegemony of the biomedical model in the current scenario, which is in contradiction with the expanded concept of health, generating an alienating and conflictive tendency in the work process that triggers professional dissatisfaction;^19^^,^^26^^,^^34^ (iv) *Formative paradigmatic*: Disparity among health education, the reality of the service and the needs of the population that require formative, innovative, problematizing and significant practices. 

Attributes: (i) *Developing a dialectical sensitivity*: Ability to observe and integrate into the context of health care, being critically sensitive to vulnerabilities and overcoming tensions, in the face of the process of communication and teaching, learning and care;^15^^,^^19^^,^^20^^,^^21^ (ii) *Promoting a dialectical attitude*: An attitude of valuing and mediating the tensions and conflicts of the teaching, learning and care process, with clarifying and emancipatory communication, transcending the panoptic character and moral judgment;^5^^,^^15^^,^^19^^,^^20^^,^^21^ (iii) *Promoting ambience*: It promotes the physical and psychological management of the environment for the promotion of care and the work process, compensating collective tensions dynamically to break bureaucratization;^20^^,^^34^ (iv) Analyzing the social determinants of health: Valuing the historical, social and political context of illness, highlighting the asymmetries and identifying barriers to an ideal health situation, exposing the contradictions that involve social actors for the promotion of accessibility and active and qualified listening.^21^^,^^26^^,^^42^

Consequents: (i) *The being who cares for and the being cared for in their social relationships*: Dialectical care provides the caregiver with the development of criticality that leads to the overcoming of alienation, identifies vulnerabilities, favors equity and promotes social justice, putting him/her in a position to transform alienating social relationships. For the being cared for, it promotes inclusion, affective bonding and empowerment;^15^^,^^21^^,^^42^ (ii) *Health services/Work process*: Valuing dialectics and overcoming vulnerabilities in the work process, promoting the professional’s emotional balance and problem-solving capacity in care. It stimulates the appreciation of historicity and the users’ life projects dialogically, as well as their personal narratives, resulting in individualized and safe care;^5^^,^^19^^,^^20^^,^^26^^,^^34^^,^^42^ (iii) *Formative paradigmatic*: The teaching of dialectical care allows exposing the contradictions between the posture of liberation and the tendency of institutionalized processes by observing the objective reality and capturing the health needs of the being cared for with the possibility of materializing genuine holistic care. 

Concept-related terms. The related terms are those pertinent to the concept of interest, but they do not share the same attributes.^6^ Both person-centered care^12^ and holistic care^17^ accompanied the concept of dialectical nursing care. Person-centered care refers to care that goes beyond the perspective of healing symptoms inscribed in the biomedical model. It is organized around the needs of the person, in a relationship of partnership between him/her, the family, the community and the health professionals. The person contributes with his/her experiences, knowledge, beliefs and preferences, while the professionals contribute with their scientific knowledge and care experiences, providing support and relevant information so that they are able to make decisions about his/her own health and manage his/her treatment.^24^^-^^26^ Holistic care involves health practices with systemic and interdisciplinary approaches, which seek the balance of the totality of the being, since it recognizes and values the interdependence between biological, psychological, social, spiritual and cultural aspects in the health-disease process.^27^ Therefore, the proposed dialectical nursing care advances beyond these two conceptions, based on the intentionally dialecticizing process, which guarantees the analysis of the health phenomenon in the social reality, where the patient moves in an alienating culture permeated by oppression, power relationships and social inequalities, in addition to proposing a synthesis for his/her overcoming and enabling transformations in himself/herself, in society and in nursing itself. 

The definition of the concept. Dialectical nursing care is characterized by the interaction between the caregiver and the being cared for based on their system of care production, their social relationships and the way in which they are historically modulated, which aims to favor the analysis and understanding of the person about the social production of his/her suffering/illness and well-being/health, maximizing his/her leading role in making health-promoting decisions and consciously managing self-care in concrete (material) reality and expanding his/her resources and social supports to overcome vulnerabilities. Dialectical nursing care has an interdisciplinary and multidimensional nature and operates in the nursing clinic through the systematic, attentive and intentional analysis of the multiple social, political and cultural contradictions that naturally cross the subject historically, in a process of problematization of reality with the being cared for, with dialectical sensitivity and attitude, which gives both the opportunity to react against the oppressions that surround him/her and to constitute syntheses and new syntheses, potentially capable of propelling him/her towards his/her relative emancipation, in the concreteness of his/her daily life, within his/her territory and to the extent of his/her desire. Its application places nursing as an articulator of an intersectoral and interprofessional care network. 

Example use of the concept (model case). Without family ties, homeless and an abusive consumer of alcohol and marijuana, hypertensive and diabetic, AML refuses to undergo multidisciplinary clinical interventions, is unable to organize himself with pharmacological treatment and has committed crimes that result in interpersonal violence and periods of judicial incarceration, which aggravate his physical and mental health. This is the panorama in which hospitalizations play an important social role, temporarily reducing his exposure to harmful agents, reestablishing his state of health in the face of clinical complications and sparing the community from his infractions, while the social and judicial assistance system forces the responsibility of family members regarding the guardianship of AML, without success, given the absence of affective ties between them. The complexity of the case has led health teams to seek intersectoral collaborative strategies, but these tend to reproduce interventions focused on containing psychiatric symptoms and providing total abstinence from substances, with psychotropic drugs and hospitalizations. These measures are bureaucratic and burdensome to the social and health system and to society, and are clearly insufficient to bring about changes in the current situation. They also generate stress and frustration among workers, who feel powerless in the face of relapses and have no social recognition for the product of their work process. Recently, a proposal for dialectical care was devised, with nursing taking the lead in intersectoral articulation, putting into perspective the social scenario and the infrastructure of the services through which AML moves, his daily life on the street and the narratives and expectations of the individual and professionals. In this context, during the shared consultation, the following items were identified: a) determinants of health: occasional support from the community for food, hygiene and protection of his movable property, cognitive condition to plan and take responsibility for the therapeutic project and agreement with the therapeutic proposal; and b) determinants of illness: stigmatization, segregation, lack of social protection, inefficiency of the technological health apparatus to meet psychosocial needs, obstruction of access to the health and social assistance system, psychiatric asylum culture, distrust of AML in the State, support from local businesses for alcohol consumption, complementary relationships with people who consume psychoactive substances, absence of structuring affective ties, clinical and psychiatric complications that reduce his availability to adhere to treatment. Thus, dialectical nursing care, based on comprehensive psychosocial strategies, which included strengthening support agreed upon in the local community and reducing harm resulting from substance consumption, recognized for its low level of demand on the person, and emphasizing the role of a family health unit, which received matrix support for technical qualification by the psychosocial care service, the intersectoral proposal restructured care in the context of the street, reaching other individuals in that vulnerable condition. Nursing has adopted new routines to facilitate access to that local audience, making examinations more flexible and allowing frequent clinical and psychological interventions, from an ethical and equitable position, adopting interpersonal communication capable of problematizing AML’s personal experiences together with him and explaining how they can contribute to his conflicts. 

The objectives included ensuring his protection, improving his health status and controlling comorbidities, thus increasing his level of knowledge to make decisions, facilitating his entry into the labor market compatible with his choices on the street, qualifying his social skills and expanding his circulation around the city, with greater contractual capacity. The actions agreed with AML began concentrate on the street, focusing on support relationships that do not concern family members or consanguinity, such as an affective companion, a local religious leader who was responsible for the storage of AML medicines and a merchant to whom AML offered occasional general autonomous services. In partnership with a philanthropic temporary shelter, AML began to obtain protection in moments of greater vulnerability, even though he refused proposals for fixed housing. Other individuals in a similar situation began to benefit from this presence of the teams, which modified their authoritarian discourses into dialectical attitudes capable of welcoming the consumption of psychoactive substances and translating it into a language denouncing social inequality and human vulnerability. It was from this change in care logic that the real demands of that audience were accepted and adherence to the proposals was noticed. Relapses, hospitalizations and violence were minimized with access to health and social assistance, but they were also naturalized by the professionals as possible experiences, but re-editable in the continuum of the health-disease process. As the health or life situation of AML changed, new agreements were made and new reorganizations of the care structure were necessary. 

## Discussion

According to Marx, the human way of existing, since the advent of capitalism, has been engendered in a conjuncture of class society that produces its culture from the transformation of nature through the work process and the construction of knowledge that can meet its demands, respond to its questions, which are crossed by irremediable contradictions that demand new syntheses and new solutions, in an uninterrupted becoming.^28^ Marx’s method of analysis stands out by situating the phenomena sought historically and socially in real life, in order to glimpse their determinants and overcome appearances, as is the case of the complaint of the individual who seeks health care. It is in dialectical historical materialism, of a Marxist nature, that dialectical nursing care is based, a product of this concept analysis.

The formulation of dialectical nursing care is justified in view of the contradictions that arise between the discourse guided by the expanded conceptions of health and contemporary health practices, which are also revealed in their biomedical, interventionist and curativist nature, which focus on the disease, the diagnostic classifications and the extinction of suffering.^11^^,^^29^ The conventional readings that are made about the phenomenon shown by the patient are guided by appearance, the clinical sign, which does not reveal the core of his suffering and his personal searches. 

The choice of a clinical case used to illustrate the dialectical nursing care is based on the clinical practice of the authors and reflects the complexity of the health-disease-care process, with people with mental disorders related to substance abuse, who are homeless. Nevertheless, it can be applied to other health contexts. Historically and socially sustained, the psychiatric institutional mechanism, which regulates human behavior, was legitimized by society to moralize and standardize the individual, and attributed to nursing a panoptic and executing function disguised as power, but which alienated it from its work process. As a result of this mechanism, we are faced with the disconnection among mental health, physical health and social services and interventions, with practices and theories strongly based on diagnostic classification systems that disregard the integration among genetic, environmental, social and neurobiological factors.^30^

The model case shows the social, cultural and clinical condition experienced by AML, which represents an effect of the capitalist model that governs Western relationships, capable of segregating and stigmatizing people. The case expresses an organic relationship between the health problem (mental disorder, its comorbidities and the harmful consumption of substances), as well as crime/conflict with the law, violence, class stratification/social inequalities implicit in the phenomenon of homelessness. Thus, nurses, in the search to implement the concept of dialectical nursing care in practice, are called upon to dialectically analyze the productivist organization in health and how it has shown a compromise in the well-being and plenitude of the population, at the same time that they are faced with the legal superstructure and how it consolidates a dialectics, by promoting a circuit of social moralization, when it penalizes delinquency and, concurrently, not reaching the determinants of social injustices, as reflected in the model case, which intends to illustrate the interface among the antecedents, attributes and consequents of dialectical nursing care. 

### The antecedents of dialectical nursing care

The antecedent “The being cared for and its social relationships” indicates relationships of marginalization, segregation and stigmatization that oppress and make individuals and groups vulnerable, thus affecting their health status.^31^ A social context thus constituted drives the revision of contemporary health practices, since the living conditions of the populations respond to social determinants of health (income, education, social protection, housing, employment, occupational safety, food safety, child development and access to quality health services) modulated by forces and systems of a political, economic and economic nature, as well as social and agreed development agendas, which entail unfair and avoidable differences in health conditions.^32^

The antecedent “Health services” denounces the incipience of the structure of the services in meeting the needs of the population and the inconsistency in the teaching-service relationship. Therefore, in the conceptions imbricated in dialectical nursing care, it is necessary to analyze the proportion in which the structure of these services compromises the provision of care, in the face of a global conjuncture of multifactorial and growing crisis and chronic and complex health conditions, which are reflected in high costs and low level of quality, with resources disproportionately allocated to demographic groups.^33^

As for the antecedent “Work process”, it reveals a prescriptive and linear care system, dictating parameters and norms that diverges from contemporary discourses on expanded conceptions of health.^34^ This reality reinforces diagnoses, group classifications and the power of pharmacological measures, while at the same time burdening public resources, making health work more precarious, emotionally exhausting professionals, chronicizing health problems and potentiating dysfunctions, thus frustrating and limiting the autonomy of assisted individuals.^31^ On the other hand, according to the Marxist perspective, the complex universe of exploitation by labor, inherent to the capitalist mechanism, generates experiences of powerlessness, estrangement and isolation, which translate into alienation.^35^ Historically, nursing is faced with the estrangement of its social relationships and its work product, when it is oppressed by the dominating medical power and when it assumes an executing and technical function, in the face of a reality of continuous confrontation in which it loses control over its work process, a condition associated with the worker’s suffering.^11^^,^^29^

The antecedent “Formative paradigmatic” shows the panopticon character of nursing education and care, which means that the behavior and bodies of nurses and nurses in training are controlled by oppressive forces resulting from the neoliberal transformation of the health system, from a sectorial education based on disciplinary curricula and hierarchical practices that symbolize dominant relationships that are expressed in society.^13^

### The attributes of dialectical nursing care

When analyzing the historical, social and political context of people in the care process, it is possible to observe and capture the asymmetries in force in the care realities, which make people respond in a unique and non-linear way to prescriptions and therapeutic projects. The attributes of dialectical nursing care intend to support the capture of the material reality of the phenomena and the nuances existing in the health context, which determine vulnerabilities of the being assisted.^36^ They are experienced in the instance of social determinations that are established in a given society. Thus, the detection of the social determinants of illness, health and care is a core attribute, which will only be achieved by nursing professionals in the experimentation of dialectical sensitivity and attitude.

Dialectical sensitivity and attitude are attributes of the concept of dialectical nursing care, which are the basis for characterizing the analysis of contradictions in a historical moment in the lives of individuals in the care model. In order to examine these attributes, it is worth resorting to the study that analyzed the concept of being sensitive in nursing and defined this phenomenon as a cognitive process of perception, attention, awareness and self-awareness, maturity and empathy in the production of good communication in which interaction is determinant. This sensitivity can vary in degree and depth, when developed and influenced, and should generate a positive result in the sense of identifying and respecting the needs of the individuals cared for.^37^. Accordingly, dialectical sensitivity is an intentional process through which nurses organize their thoughts, using the faculty of feeling and developing dynamic syntheses to favor the overcoming of contradictions that affect the health and disease process.^38^

In a complementary way, professional attitudes are mentally produced, at a conscious or unconscious level, culturally learned and organized by experience, and imply values, beliefs or feelings that predispose the professional to make decisions.^39^^-^^41^ The dialectical attitude is an attribute loaded with values and conceptions guided by the historical and dialectical paradigm and by the social production of health, which impacts on nursing intervention,^42^^,^^43^ intending to expand the clinic, by naturalizing for the professional a reading of society based on the relationships of production, consumption and power, which are determinants of the human way of living and that define its own consciousness,^28^ where the being cared for is called to the scene as a protagonist, capable of dialectically analyzing his/her life context, making decisions and provoking transformations.

Ambience is another attribute of dialectical nursing care, functioning as a facilitator for the development of dialectical sensitivity and attitude. It is related to personnel management, well-being in the service, the physical area available for care, the necessary equipment and instruments, and the existence of indicators that allow the model that is being operated for care to be guided. The environment supports nursing care with structural elements of the services that strengthen the communicative act between nurses and the assisted individuals, and is unveiled as a correlational attribute with the work process.^44^ Therefore, ambience is not reduced to the analysis of the material structure of services, as it provides individuals and the community with sensations that go beyond physical comfort, capable of developing psychological and social well-being.^45^

### The consequents of dialectical nursing care

As regards the consequent “The being who cares for and the being cared for in their social relationships”, it can be inferred that the insertion of the person, family and community in the social fabric in a conscious way, visualizing how the social determinants of health appear and act in their habitat, allows forging possibilities of new responses that tend to affect their development and health status. In a model of care conducted in this way, individuals are instrumentalized to transcend processes of submission and alienation, thus overcoming contradictions based on renewable syntheses. The probable satisfaction in this experience opens up the possibility for the analysis of new contradictions and, so on, in a horizon of overcoming.^5^

The consequent “Health services/Work process” indicates the way to overcome the programmatic vulnerability of the health system, from the moment it makes explicit the organizational ambivalences and provides opportunities for resistance and protagonism of civil servants in the political sphere, thus enhancing structural changes. Accordingly, dialectical analysis promotes advances in the work process by inciting different interpretations of a material reality of the nursing category in constant movement, not imposed and immutable, taking into account health practices, the ideal and the possible, as well as different perspectives of workers’ health.^46^

The consequent “Formative paradigmatic” indicates that the conceptual development of a historical and dialectical care structure in nursing education proposes the revision of competencies, skills and attitudes necessary for the compatibility between service/professional and the needs of individuals/society, and that they focus attentively on multidimensional issues of the health-disease process. It refers to dialectical learning, which is based on the interaction between person and environment as fundamental element for the production of knowledge, which explores, during formative practice, the tensions and opposites that are constitutive of dynamic scenarios in real life.^47^^,^^48^ The formative practice of a dialectical nature exposes educators and students to situations of resistance and changes, including the organizational nature of the curriculum in its philosophical pedagogical references, which lead to alternations of results, assuming that one social arrangement gives way to another, successively.^48^ In this field, the idea and ideal of care under the reference of the social production of health is permanently analyzed in terms of the interpenetration of opposites, that is, in terms of intertwining the elements of reality, which cannot be analyzed in isolation.^48^

The nursing interventions produced in the model case were formulated by considering the individual in his/her existence-suffering, and the dialectical historical materialist approach is a possibility to consider the human being in a changing historical context, in order to overcome the focus on the disease, the biological dimension and the medicalization of problems.^11^

Regarding the implications for nursing, what the praxis support of the concept of dialectical nursing care tends to operate in nursing professionals is resistance against dominating structures and forces and the freedom to direct their work process, in line with their ethics and deontology, bringing to the nursing professional’s awareness the extensive range of dimensions belonging to care, from what the person and his/her environment reveal and from what he/she weaves, which unveils other problems and other possibilities of solution, in the continuum of the health-disease-care process. Historically, humanistic forms of care inscribed in psychosocial models expand in relation to biomedical practices and express values of the extended clinic, turning to the subject in suffering and valuing his/her surroundings in the care process, in actions chained in the territory and that integrate the subject into his/her culture.^11^ The contribution of the concept of dialectical nursing care is to clarify and standardize a language about this approach to care, which highlights the ambivalences that are established in the scope of the social production of health, promoting careful investigation from the dialectical historical perspective, capable of sharpening the caregiver’s dialectical sensitivities and attitudes, which allow the development of dynamic care practices, renewable and surmountable with each care process.

As for the limitations, the current study was based on Rodgers’ concept analysis method, which requires a literature review, without, however, having a review system. Nonetheless, an integrative review was chosen. In addition, the search for articles was restricted to the following languages: English, Portuguese and Spanish. 

Conclusion. The production of the concept of dialectical nursing care was developed based on Rodgers’ Concept Analysis and was based on antecedents captured in human and social relationships, in the functionality of health services and their work processes, as well as in training. In these analysis scenarios, the authors sought the motivations to rethink a care model. Both in the social determinations in health and in the promotion of the environment in health services, the attributes of dialectical care expose the driving elements of the dialectical sensitivity and attitude developed and improved in nursing professionals. The consequents resulting from dialectical nursing care are potential improvements in the response of services to the needs of the population and qualification of the relationship between the being cared for and society, in the structure of services and in the relationship between the latter and professionals. In addition, it tends to break educational paradigms. In this way, the analysis of the dialectic inscribed in the social production of suffering/illness and well-being/health is fostered, maximizing people’s protagonism, in order to make health-promoting decisions compatible with the social reality and historical moment, operating syntheses and new syntheses. It is considered that this concept, based on evidence, drives nurses to overcome paradigmatic barriers and challenges, with a macrosocial, formative and procedural dimension of work. Ensuring healthy lives and promoting well-being for all, at all ages, is one of the challenging goals of the United Nations and its member states to meet by the year 2030 in achieving the Sustainable Development Goals (SDGs). The concept of dialectical nursing care aims to respond to SDG 3, which intends to promote the mental health and well-being of all people, at all ages. New technologies for promoting care become fundamental to achieve this objective. Furthermore, this concept was investigated from the perspective of the nursing literature; however, the social and interdisciplinary dimension of dialectical care foresees intersectionalities that suggest its multiprofessional incorporation.
